# Debranching enzymes decomposed corn arabinoxylan into xylooligosaccharides and achieved prebiotic regulation of gut microbiota in broiler chickens

**DOI:** 10.1186/s40104-023-00834-3

**Published:** 2023-03-09

**Authors:** Wei Wu, Huajin Zhou, Yanhong Chen, Yuming Guo, Jianmin Yuan

**Affiliations:** State Key Laboratory of Animal Nutrition, College of Animal Science and Technology, China Agricultural University, 2 Yuanmingyuan West Road, Haidian District, Beijing, 100193 PR China

**Keywords:** Arabinoxylan, Corn, Prebiotic, Specific xylanase, Xylooligosaccharide

## Abstract

**Background:**

Corn arabinoxylan (AX) is a complicated and multibranched antinutritional factor, thereby proving the use of endo-xylanase (EX) to be marginally valid. This study focused on specific types of AX-degrading enzymes (ADEs) to exert the synergy of debranching enzymes and track the prebiotic potential of enzymatic hydrolysates. This study investigated the effects of ADEs on the growth performance, intestinal histomorphology, absorption functions, changes in polysaccharide components, fermentation, and gut microbiota of broiler chickens. Five hundred seventy-six five-day-old Arbor Acres male broiler chickens were randomly allocated into eight treatments with six replicates each. Corn basal diets supplemented with or without enzymes were fed for a 21-day period, specifically including EX, its compatible use with arabinofuranosidase (EXA) or ferulic acid esterase (EXF), and compound groups with the above three enzymes (XAF).

**Results:**

Specific ADEs stimulated the jejunal villus height and goblet cell number and evidently decreased the crypt depth (*P* < 0.05), while the ratio of ileal villus height to crypt depth was significantly increased in EXF (*P* < 0.05). Maltase activities of ileal mucosa in XAF groups were extremely enhanced (*P* < 0.01), and EX boosted the activity of Na^+^-K^+^ ATPase in the small intestine (*P* < 0.01). The insoluble AX concentrations comparatively lessened, thereby notably raising the sundry xylooligosaccharide (XOS) yield in the ileal chyme (*P* < 0.05), which was dominant in xylobiose and xylotriose. Improvements in the abundance and diversity of ileal microbial communities within the EXA, EXF, and XAF treatments were observed (*P* < 0.05). Positive correlations between microbiota and XOS were revealed, with xylobiose and xylotriose being critical for ten beneficial bacteria (*P* < 0.05). EXF increased the BWG and FCR of broiler chickens in this phase (*P* < 0.05), which was attributed to the thriving networks modified by *Lactobacillus*. The intracecal contents of acetic acid, butyric acid, and propionic acid were greatly enhanced in most ADE groups, such as EXF (*P* < 0.05).

**Conclusions:**

Debranching enzymes appreciably targeted corn AX to release prebiotic XOS in the posterior ileum and facilitated intracaecal fermentation. It was beneficial for improving gut development, digestion and absorption and modulating the microflora to promote the early performance of broiler chickens.

**Graphical Abstract:**

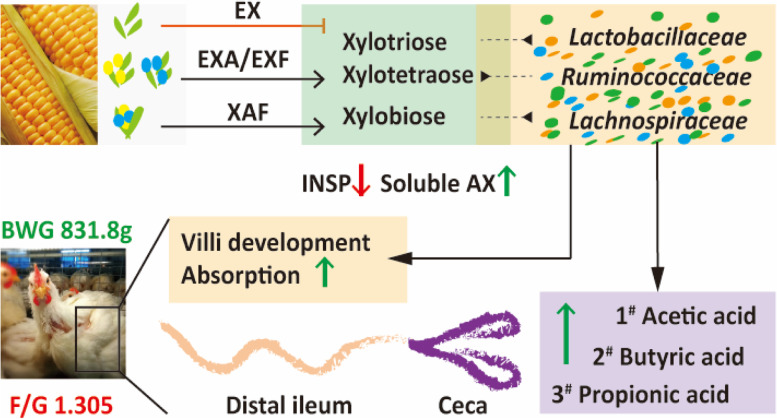

**Supplementary Information:**

The online version contains supplementary material available at 10.1186/s40104-023-00834-3.

## Introduction

The relatively complex gut microecosystem plays an indispensable role in maintaining intestinal health, and the diversifications in microbiota are inseparable from dietary factors [1]. The common nonstarch polysaccharides (NSPs) in the diet, especially grain-derived arabinoxylan (AX), are inclined to increase the viscosity of chyme in the intestinal lumen and disturb the normal microflora [2]. This can result in reductions in the nutrient utilization efficiency and growth performance of the host. Although exogenous endo-xylanase (EX) eliminates the negative effects of AX to a certain extent, its efficacy can be abridged by factors such as the source of enzyme-producing strains, the expressed enzyme activities, the inclusion levels, and the experimental animals. Furthermore, due to the specificity of enzymes, the structure of the degraded substrate is of paramount importance. A higher frequency of branched attachments and substituent groups hinders enzymatic cleavage of the AX backbone [3]. Constructed as such, it might require a battery of enzymes projected not to differ so much in the core EX but rather in conjunction with debranching enzymes to attack side chains or indestructible linkages [4].

An abundance of data are focused on wheat-based grains, where efficacy for both in vitro and in vivo settings has been varied [5, 6]. Additionally, solitary EX has proven to have limited validity. Conversely, our previous study systematically demonstrated that specific arabinoxylan-degrading enzyme (ADE) collaborations among EX, arabinofuranosidase (EA), and ferulic acid esterase (EF) increased AX degradation to more oligomers, which improved the growth performance and gut health of broilers fed wheat diets [7–9]. However, corn, a nearly universal ingredient in poultry feed, retains structural complexity and varies considerably in AX solubility compared with wheat. There is a lack of insight reporting the potential for specific ADE synergism on typical corn diets. Relevant reports have emphasized the in vitro solubilization or fermentation of corn AX by these carbohydrases [10, 11], as opposed to in-depth tracking and monitoring of their potential in vivo effects, especially involving gut microbiota or microecosystems. Based on the synergistic mechanism of the debranching enzymes and the main enzyme EX, we hypothesize that it may require diverse combinations or even multiple doses of specific ADE preparations for corn-type basal diets. Moreover, greater attention needs to be paid to whether there are certain differences in the enzymatic hydrolysis products of corn AX, and the product components are beneficial to maintain intestinal health.

The formation of xylooligosaccharides (XOS) is recognized as a merit of AX degradation, in which xylobiose and xylotriose are the more potent functional components. Scholars compared the prebiotic effects and fermentation abilities of five commonly used fibers through in vitro system measurements [12] and found that XOS significantly increased the abundance of *Bifidobacterium* after 24 h and promoted the formation of short-chain fatty acids (SCFAs). *Lactobacillus plantarum* S2 had higher cell density and faster growth rates when cultured on corncob-derived XOS, and acetate was the predominant SCFA in the final fermentation products. Much attention has also been devoted to their prebiotic roles in mediating intestinal homeostasis and maintaining the mutually beneficial relationship among microbes [13–15]. The in situ production of corn XOS is of great significance to the regulation of intestinal microflora. However, there is a lack of understanding about the AX conversion, as well as the different sizes and quantities of XOS fractions (xylobiose, xylotriose, xylotetraose, xylopentose, etc.) manufactured in the gastrointestinal tract as a result of feeding birds basal diets supplemented with specific ADEs. The scientific doubt needs to be answered in combination with the analysis of intestinal content products and microbiome technology.

Collectively, this study aimed to evaluate the effects of different specific ADE combinations or inclusion levels on the growth performance, intestinal morphology, digestive enzyme activities, enzymatic hydrolysis product composition, fermentation metabolism, and gut microbiota of broiler chickens via a feeding trial. Allowing for the possible clarification of the differential regulation of the enzymatic hydrolysis products of corn-type AX on the endogenous intestinal probiotics of broiler chickens and providing a solid theoretical foundation for the improvement of intestinal health and application promotion of prebiotic ADEs.

## Materials and methods

### Ethical statements

The animal experimental design and procedures were approved and conducted under the supervision of the China Agricultural University Laboratory Animal Welfare and Animal Experimental Ethical Committee (No. AW80011202–1-3) (Beijing, China).

### Enzyme preparations in the animal trial

The *Aspergillus niger*-derived endo-xylanase (EX), arabinofuranosidase (EA), and ferulic acid esterase (EF) (90,000, 10,000, and 1000 U/g, respectively) were provided by Bestzyme Bio-products, Co., Ltd. (Jinan, China). EX and two other debranching enzymes were dried powder samples that had been subjected to tolerance assessment and enzyme activity assays.

### Experimental design, diets, and management

In total, 576 5-day-old Arbor Acres male broiler chickens were weighed and randomly allocated to eight treatments with six replicates of twelve chicks each so that their initial body weights were similar across all the groups. The birds were fed a basal diet as the control treatment group (CTL), whose composition and nutritional levels are shown in Table 1, and the corresponding diets supplemented with specific arabinoxylan-degrading enzymes (ADEs) (Table 2). Briefly, single xylanase (EX), its compatible addition with EA (EXA) or EF (EXF), and compound groups with the above three enzymes (XAF-1, 2, 3, 4) were included. Through the multi-stage and step-by-step premixing procedure, the enzyme preparations of each treatment were reasonably supplemented into the corn basal diet of broiler chickens. Supplemental enzyme levels were based on the in vitro screening results [16]. Diets were fed in mash form (passed through a 3.60-mm sieve screen). The growth trial lasted for 21 d. All chicks were reared in cages (100 cm long × 80 cm wide × 40 cm high) with nipple drinkers, and the house was environmentally controlled at standard conditions of temperature and ventilation. The lighting schedule was 20 h light and 4 h dark throughout the period. Feed and fresh water were available ad libitum. Birds were vaccinated using combined Newcastle disease virus and infectious bronchitis virus on 7 d via intranasal and intraocular administration.

**Table 1:** Composition and nutrient levels of corn basal diet (as-fed basis)

Items, % for units unless noted	d 1 to 21
Ingredients	
Corn (7.8%, crude protein)	51.50
Soybean meal (46%, crude protein)	30.58
DDGS^a^ (28%, crude protein)	5.00
Corn gluten meal (63.5%, crude protein)	4.00
Wheat flour^b^	2.00
Soybean oil	2.40
Limestone	1.27
Sodium chloride	0.35
Dicalcium phosphate	1.94
Choline chloride (50%)	0.20
*DL*-methionine (98%)	0.24
*L*-lysine·HCl (99%)	0.26
Antioxidant	0.02
Multivitamin^c^	0.03
Multimineral^d^	0.20
Phytase (10,000 U/g)	0.01
Total	100.00
Calculated nutrient levels	
Metabolic energy, Mcal/kg	2.95
Crude protein	22.5
Available phosphorus	0.45
Calcium	1.00
Lysine	1.30
Methionine	0.59
Threonine	0.85
Tryptophan	0.25

^a^DDGS, distillers dried grains with solubles

^b^Adding flour to the basal diet was done to improve pelleting quality

^c^Supplied per kilogram of diet: retinyl acetate, 9500 IU; cholecalciferol, 2500 IU; α-tocopherol acetate, 30 IU; menadione, 2.65 mg; thiamin, 2 mg; riboflavin, 6 mg; cyanocobalamin, 0.025 mg; biotin, 0.0325 mg; folic acid, 1.25 mg; pantothenic acid, 12 mg; and niacin, 50 mg

^d^Supplied per kilogram of diet: copper, 8 mg; zinc, 75 mg; iron, 80 mg; manganese, 100 mg; selenium, 0.15 mg; and iodine, 0.35 mg

**Table 2:** Treatments with specific arabinoxylan-degrading enzymes and relevant inclusion levels in animal trial^a^

Treatments	CTL	EX	EXA	EXF	XAF-1	XAF-2	XAF-3	XAF-4
EX	0	54 **(600)**	54 **(600)**	54 **(600)**	54 **(600)**	63 **(700)**	63 **(700)**	54 **(600)**
EA	0	0	5 **(500)**	0	5 **(500)**	6 **(600)**	4 **(400)**	6.7 **(668)**
EF	0	0	0	0.4 **(400)**	0.4 **(400)**	0.5 **(500)**	0.5 **(500)**	0.4 **(400)**

^a^Treatments: CTL, basal diet (control); EX, basal diet supplemented with core xylanase; EXA, basal diet supplemented with xylanase accompanied by EA; EXF, basal diet supplemented with xylanase accompanied by EF; XAF-*i* (*i* = 1–4), compound groups with the above three enzymes. The same as below, also involving all figures. Bold values in parentheses are additive dosages (mg/kg) in this animal experiment, while the values outside brackets are the appropriate inclusion levels (U/g) obtained from the in vitro screening process

### Growth performance

Body weight and feed consumption were recorded for each replicate at 21 d. Thus, body weight gain (BWG), along with feed intake (FI), and the feed-to-gain ratio (F/G) during the starter period were calculated. Moreover, the mortality (MRT) and European comprehensive production index (EPI), based on the death and elimination of broiler chickens in this stage, were calculated. Specifically, EPI was calculated by the following formula:

$$\textrm{EPI}=\textrm{100}\times \textrm{ABW}\times \textrm{LB}/\left(\textrm{F}/\textrm{G}\times \textrm{D}\right)$$

where EPI is the abbreviation of European comprehensive production index; ABW is the average body weight of live broilers at 21 d (kg); LB represents the livability of broilers in this stage (%); F/G is feed to gain ratio of broilers in this stage (g/g); D represents the number of days.

### Slaughter method and sample collection

At 21 days of age, six healthy chicks from each treatment were bled from the carotid artery after being anesthetized by an injection of sodium pentobarbital (50 mg/kg body weight) in the wing vein. Then, mid-segments of the jejunum and ileum at necropsy were immediately removed and fixed in 4% paraformaldehyde solution for intestinal morphology measurements [17]. Digesta and mucosa from the remaining intestinal portions and ceca were separately harvested and stored at − 80 °C for further analysis.

#### Intestinal histomorphology and absorption function

Five-micrometer consecutive sections of jejunal and ileal segments were prepared for morphological observations after staining with hematoxylin-eosin (HE). Ten representative and well-oriented villi and the associated crypt of each sample were selected for morphological observations using a Leica DMi8 optical microscope (Leica Corp., Weztlar, Germany). Villus height (VH) was ascertained by measuring distance from the apex of the villus until the junction of the villi and crypt [17]. Crypt depth (CD) was defined as the depth between the villus and the basal membrane. Accordingly, the villus height to crypt depth ratio (VCR) was calculated. Moreover, ten intact and neat rows of intestinal villi stained with periodic acid Schiff (PAS) were selected to image goblet cells (GC). The number of GC was quantified by counting the number of stained goblet cells per 100 μm length of villi and presented as the means per ten villi. The activities of mucosal sucrase, maltase, alkaline phosphatase, and Na^+^-K^+^ ATPase were determined colorimetrically via commercial kits (Nanjing Jiancheng Bioengineering Institute, Nanjing, China) according to the manufacturer’s protocols. These indices were normalized by the total protein contents of the intestinal mucosa, which were quantified using bicinchoninic acid (BCA) protein assay kits (CWBiotech Co. Ltd., Beijing, China). Specific principles and operating procedure were inquired through the instruction manual of the kits.

#### Analysis of NSP in the distal ileum chyme of broiler chickens

The soluble and insoluble NSPs or xylan contents were measured as newly illustrated with minor modifications [18]. Ileal chyme samples were pretreated with fat extraction and enzymatic hydrolysis of starch. Subsequently, the supernatant and residue were subjected to different complicated steps such as hydrolysis, washing, centrifugation, and drying. The glycan degradation products were then analyzed for individual sugar concentrations by high-performance liquid chromatography (UPLC, Agilent 1200 series, Agilent Technologies, Santa Clara, CA, USA); the quantity of arabinose and xylose determined the AX content, and the total sugars represented the total NSP content. Monosaccharide standards consist of galactose (Gal), glucose (Glu), mannose (Man), arabinose (Ara), xylose (Xyl), fucose (Fuc), rhamnose (Rha), galacturonic acid (Glc), and glucuronic acid (GlcA) (Sigma-Aldrich Chemical Co., St. Louis, MO, USA), which were subjected to the same procedures as the samples.

#### Analysis of XOS in the distal ileum and cecal chyme of broiler chickens

The XOS contents were evaluated by high-performance anion-exchange chromatography (HPAEC). Samples were dissolved in ultrapure water, oscillated by ultrasound, and then centrifuged at 6000 × *g* for 15 min, and 1 mL of the supernatant was collected. Standards of xylobiose (X2), xylotriose (X3), xylotetraose (X4), xylopentaose (X5), and xylohexaose (X6) were purchased from Megazyme (Wicklow, Ireland, UK). The following steps were conducted according to the method [18]. Analysis of the standards and filtered samples was carried out on a Dionex ICS3000 system equipped with a pump and an amperometric detector. The chameleon chromatography management system (Dionex, Sunnyvale, CA, USA) was used for sugar identification and quantification. An analytical CarboPac PA10 pellicular anion-exchange resin column (250 mm × 4 mm) was used for sugar separation. The monoses were eluted with 250 mmol/L NaOH at a flow rate of 1.0 mL/min.

#### Bacterial 16S rDNA sequencing of ileal microbiota

The concentration and purity of total genomic DNA, which was extracted from ileal digesta following the CTAB method, was monitored by 1% agarose gel electrophoresis. 16S rDNA sequences spanning the distinct regions V3-V4 were amplified with the primer sets 515 F and 806 R with barcodes. All procedures were conducted by Novogene Bioinformatics Technology Co. Ltd. (Beijing, China) as described previously [19]. Beta diversity was visualized by multivariate statistical methods such as principal coordinates analysis (PCoA) and non-metric multidimensional scaling (NMDS). Visual heat maps were acquired according to the Spearman correlation and its significance between microbial species and intestinal apparent index. Redundancy analysis (RDA) and variance partial analysis (VPA) were adopted to quantify interpretations of the distribution of microbial communities by certain factors. Graphviz was used to draw the microbial interaction network for each treatment considering the species abundance and correlation between each genus.

#### Determination of SCFA concentrations in the ceca

Referring to the detailed method and process described by Wu et al. [17], the composition and concentration of SCFAs, such as formic acid, acetic acid, propionic acid, butyric acid, valeric acid, isobutyric acid, and isovaleric acid, were used as standards and were analyzed by gas chromatography SCION 456-GC (SCION Instruments, Goes, the Netherlands) with a flame ionization detector.

### Statistical analysis

One-way ANOVA and Duncan’s test for multiple comparisons were employed to identify differences in this trial (IBM SPSS statistics software version 21). A probability of *P* < 0.05 was considered statistically significant, *P* < 0.01 was described as extremely significant, and 0.05 < *P* < 0.10 was defined as a tendency towards significance.

## Results

### Specific ADE improved the morphology and absorption of the small intestine in broiler chickens

**Fig. 1:**
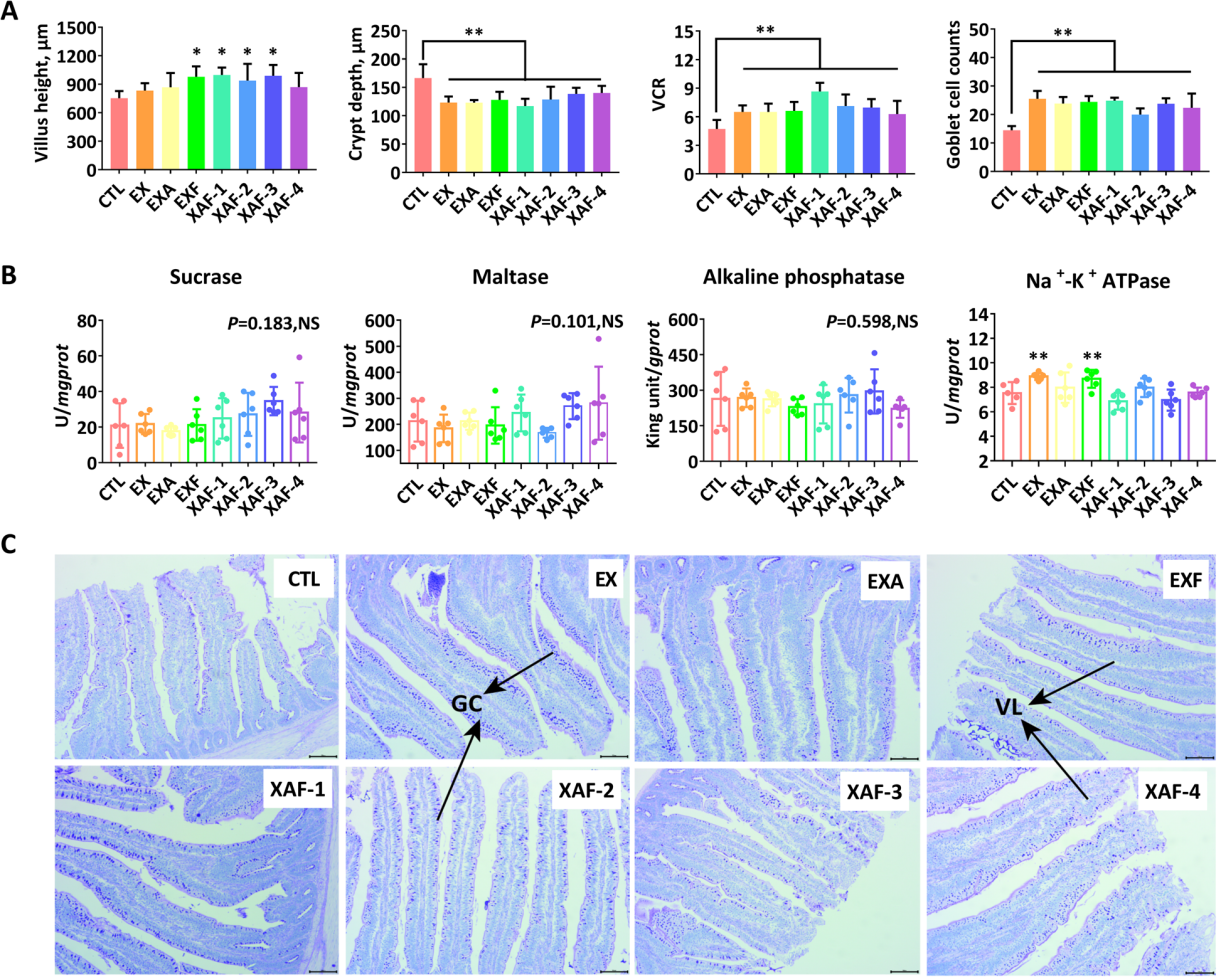
Intestinal histomorphology, digestion, and absorption functions of the jejunum in broiler chickens. **A** VH, villus height, μm; CD, crypt depth, μm; VCR, the ratio of VH to CD; goblet cell (GC) counts between each set of per 100 μm intestinal mucosal epithelial columnar cells; **B** Activities of digestive enzymes in jejunal mucosa; **C** HE-stained sections were monitored by optical microscopy at 200 × for differences in villi (VL) length and quantity of GC. Bars labeled with asterisks are significantly different (^*^P < 0.05, ^**^P < 0.01) compared with CTL. NS, no significance (*P* > 0.05). Data are expressed as the means ± SEM (*n* = 6)

**Fig. 2:**
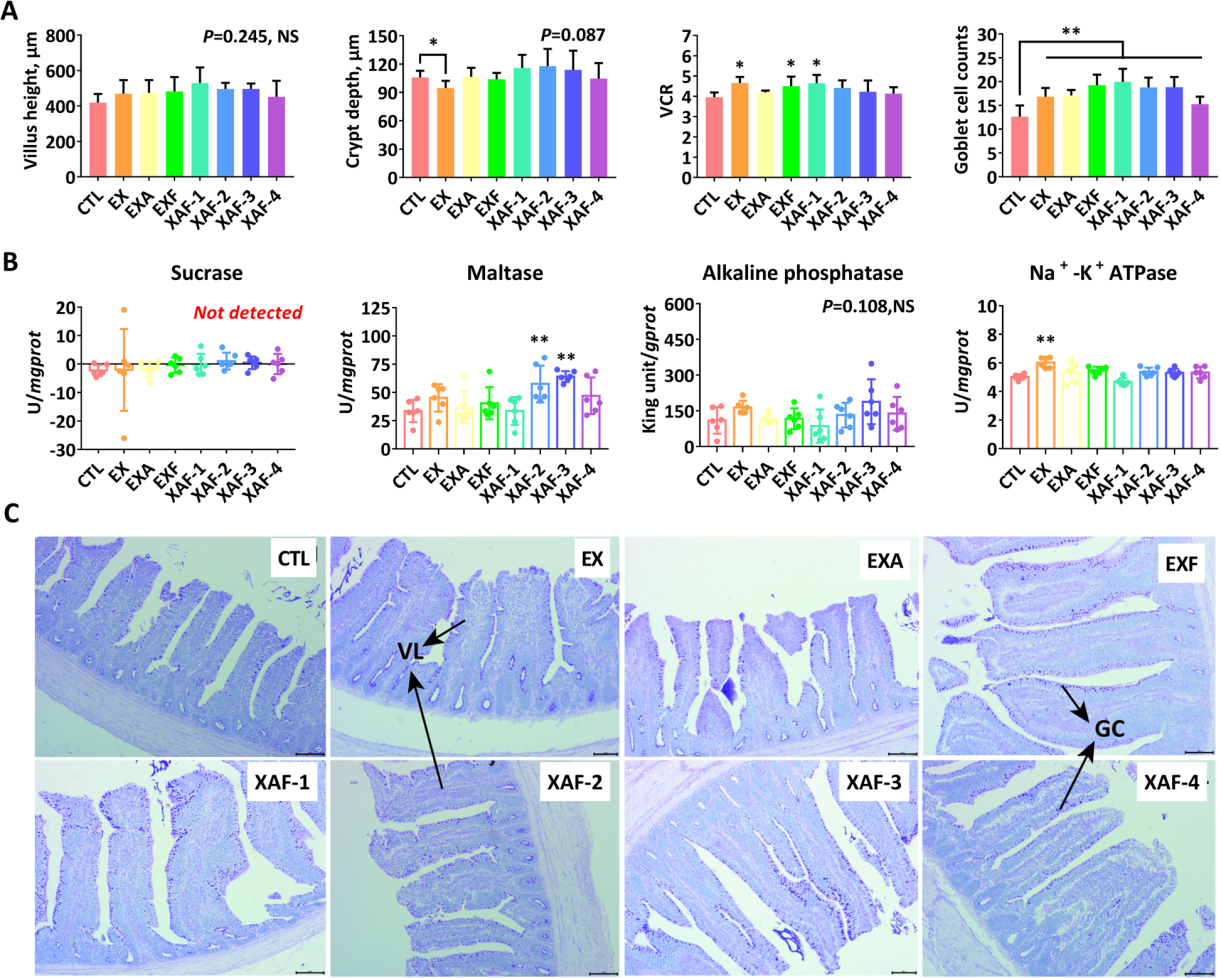
Intestinal histomorphology, digestion, and absorption functions of the ileum in broiler chickens. **A** VH, villus height, μm; CD, crypt depth, μm; VCR, the ratio of VH to CD; goblet cell (GC) counts between each set of per 100 μm intestinal mucosal epithelial columnar cells; **B** Activities of digestive enzymes in ileal mucosa; **C** HE-stained sections were monitored by optical microscopy at 200 × for differences in villi (VL) length and quantity of GC. Bars labeled with asterisks are significantly different (^*^P < 0.05, ^**^P < 0.01) compared with CTL. NS, no significance (*P* > 0.05). Data are expressed as the means ± SEM (*n* = 6)

As shown in Fig. 1A, specific ADE combinations, except EX, EXA, and XAF-4, significantly increased the VH of the jejunum at 21 d (*P* < 0.05). All enzyme treatments were demonstrated to significantly stimulate (*P* < 0.01) the jejunal VCR and goblet cell counts of broiler chickens and reduce the corresponding CD (Fig. 1A, C). Specifically, EX, EXF, and XAF-1 equally resulted in higher VCR (*P* < 0.05) and more GC quantities (*P* < 0.01) in the ileum (Fig. 2A, C). Broiler chickens fed the XAF-2/3 diets had greater (*P* < 0.01) maltase activities in the ileal mucosa (Fig. 2B), while EX and EXF extremely upregulated (*P* < 0.01) the Na^+^-K^+^ ATPase activities in the small intestinal mucosa at 21 days of age (Figs. 1B and 2B).

### Specific ADE promoted the partial enzymatic hydrolysis of corn AX to XOS in the ileum of broiler chickens

**Fig. 3:**
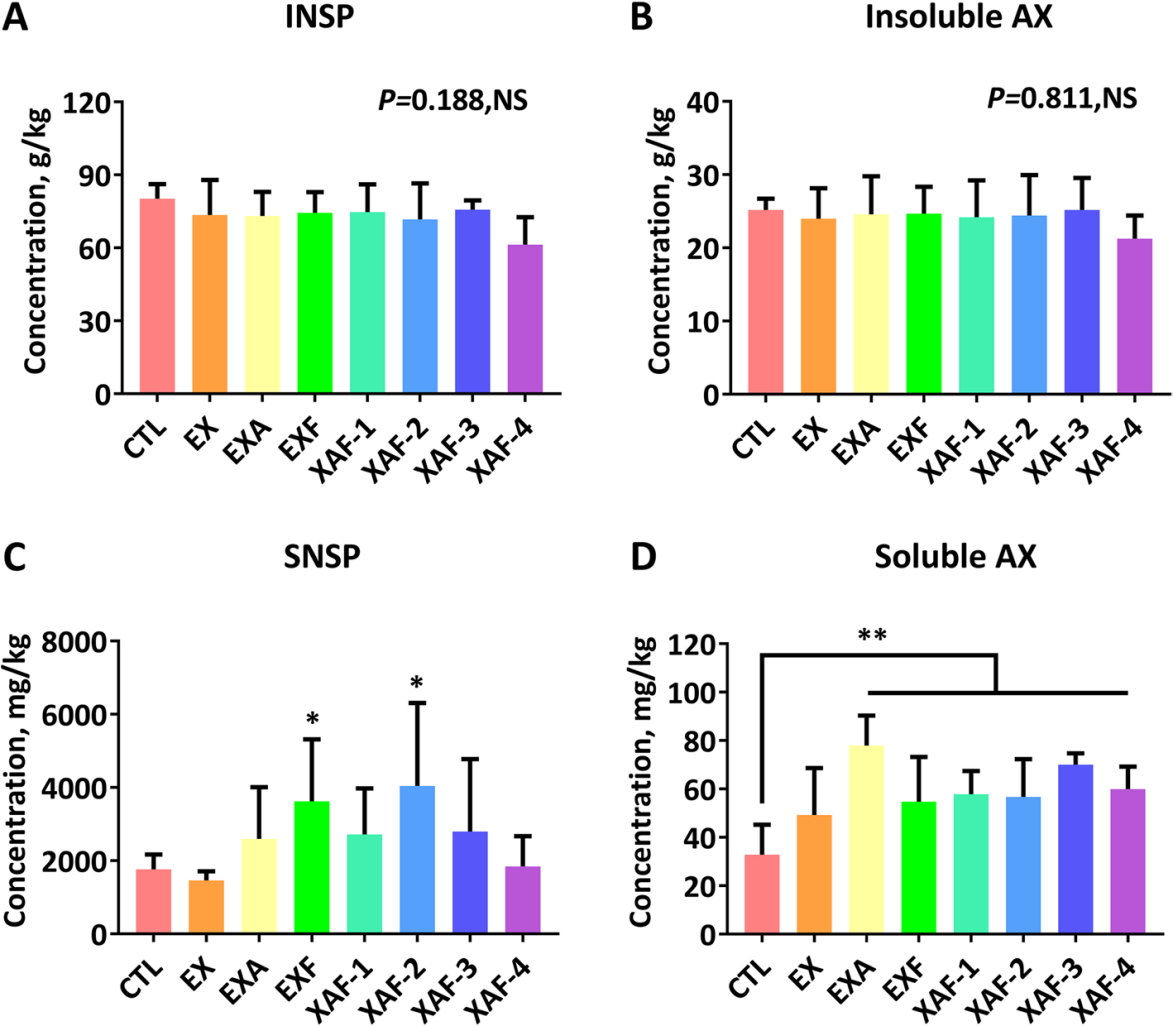
Polysaccharide changes in broiler chickens ileal chyme treated with specific arabinoxylan-degrading enzymes. Contents of insoluble nonstarch polysaccharides (INSP) (**A**) or AX (**B**), and the soluble nonstarch polysaccharides (SNSP) (**C**) or AX (**D**). Bars labeled with asterisks are significantly different (^*^P < 0.05, ^**^P < 0.01) compared with CTL. NS, no significance (*P* > 0.05). Data are expressed as the means ± SEM (*n* = 6)

**Fig. 4:**
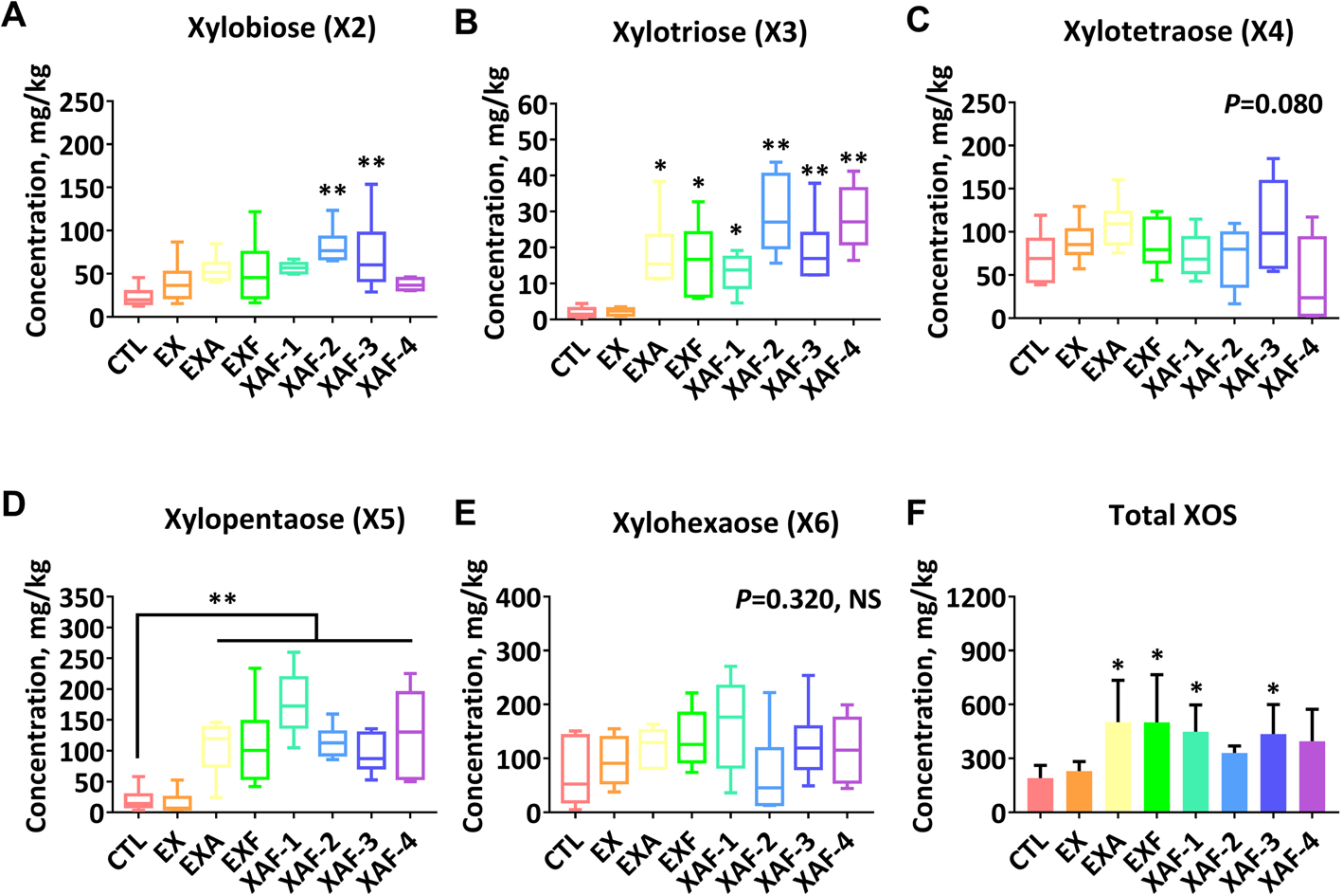
XOS production in broiler chickens ileal chyme treated with specific arabinoxylan-degrading enzymes. Various XOS components, such as xylobiose (**A**), xylotriose (**B**), xylotetraose (**C**), xylopentaose (**D**), xylohexaose (**E**), and the total output of XOS (**F**). Bars labeled with asterisks are significantly different (^*^P < 0.05, ^**^P < 0.01) compared with CTL. NS, no significance (*P* > 0.05). Data are expressed as the means ± SEM (*n* = 6)

There were extremely significant hoists (*P* < 0.01) for soluble arabinoxylan (SAX) concentration in the chyme among the treatments except for the EX group (Fig. 3D). Broiler chickens in the EXF and XAF-2 groups exhibited higher (*P* < 0.05) concentrations of soluble NSP (SNSP) (Fig. 3C). The amount of insoluble arabinoxylan (IAX) and INSP consistently decreased (*P* > 0.05) in birds fed ADE compared with those in the CTL group (Fig. 3A, B). Moreover, supplementation with XAF-2/3 significantly enhanced (*P* < 0.01) the concentration of X2 (Fig. 4A), whereas much higher concentrations of X3 and X5 (*P* < 0.01) were detected in enzyme treatments other than EX (Fig. 4B, D). The EXA, EXF, and XAF-1/3 groups notably increased (*P* < 0.05) the total xylooligosaccharide (T-XOS) content of the ileal chyme at 21 d (Fig. 4F). Intuitively, the complex of a single xylanase and a specific debranching enzyme (EXA) dominated in X4, while the three-enzyme complex treatments (XAF) concentrated on X2 and X3 (Fig. S1). In contrast, all the components and proportions of XOS in the EX group were close to those in the CTL group (Fig. S1).

### Specific ADE modulated the gut microbiota of broiler chickens through the prebiotic effects of its enzymatic hydrolyzate XOS

**Fig. 5:**
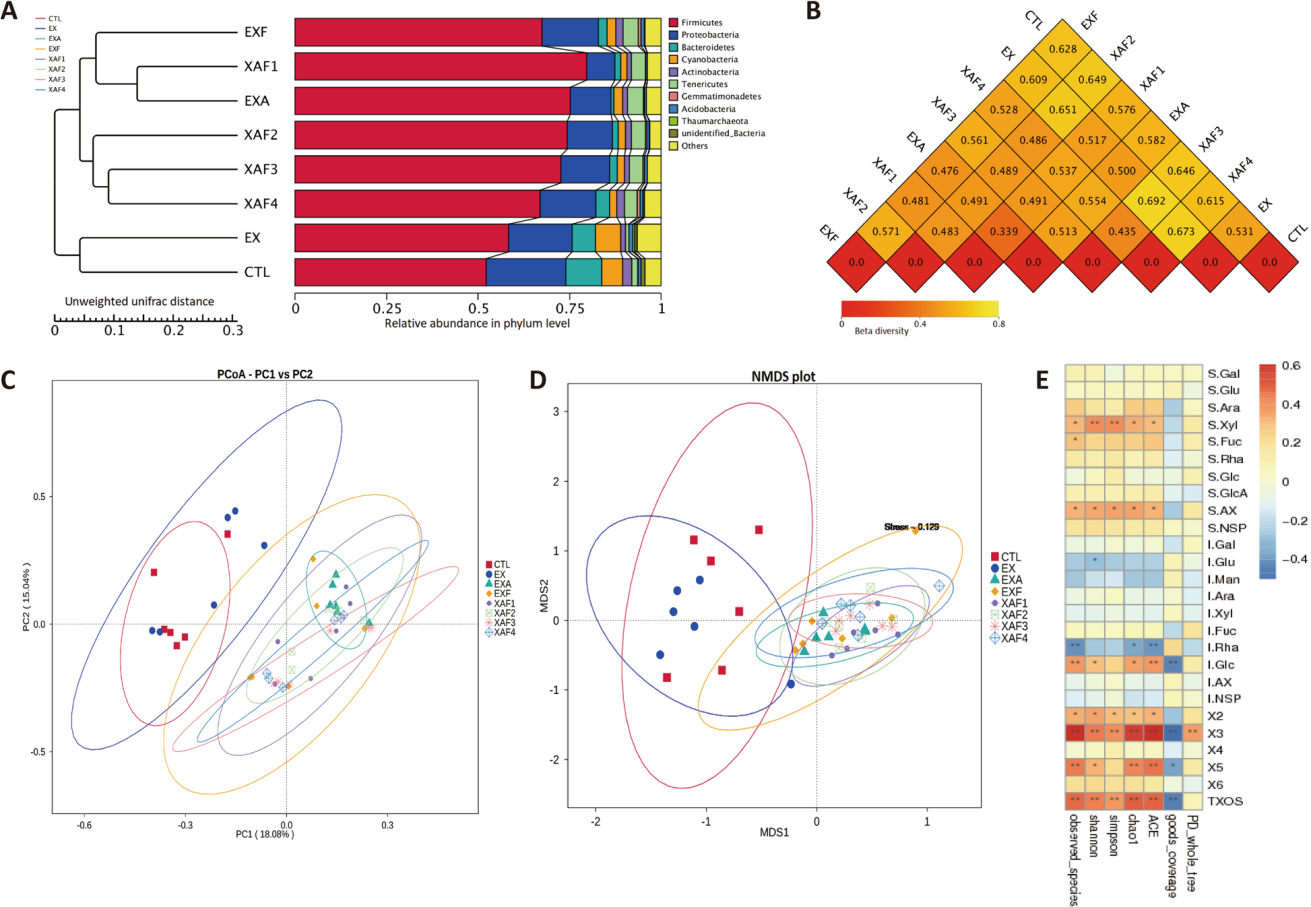
Specific arabinoxylan-degrading enzyme supplementation shifted the ileal microbiota community of broiler chickens. **A** Similarity among samples were clustered by UPGMA; **B** Difference coefficients between two treatments were measured based on the unweighted unifrac distance; **C**, **D** Disparity of ileal microbiota structure by PCoA and NMDS analysis; **E** Spearman’s association map (red, positive correlation; blue, negative correlation) integrating the microbial diversity with changes of carbohydrate composition (^*^ *P* < 0.05, ^**^ *P* < 0.01)

**Fig. 6:**
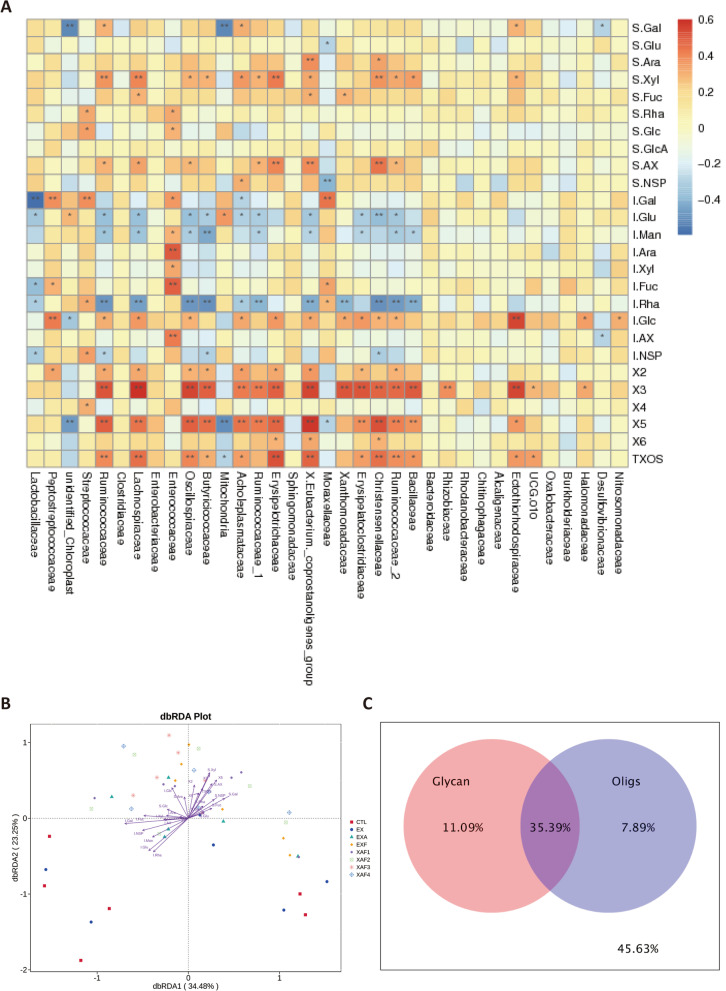
Correlation between XOS and ileal microbial constitution after treatment with specific arabinoxylan-degrading enzymes. **A** Heatmap of Spearman’s correlation coefficients between carbohydrate composition and the family level of ileal microbiota. The intensity of the colors points to the degree of associations (^*^P < 0.05, ^**^P < 0.01). S., soluble sugars; I., insoluble sugars; **B** dbRDA analysis of environmental variables (XOS) based on OTUs; **C** Influences of glycan variables and oligosaccharide products (Oligs) on the bacterial community composition via variation allocation analysis

Overall, alpha diversity parameters reflecting the microbial richness and evenness sharply increased after supplementation with EXA, EXF, XAF-1, and XAF-2 (*P* < 0.01) (Fig. S2); specifically, the EXF group had the most unique OTUs (Fig. S2F). According to the composite analysis of UPGMA, PCoA, and NMDS, the community structures from different specific ADEs occupied distinct positions, except for EX which was clustered with the CTL group (Fig. 5A, C, D). Likewise, it was consistent with the higher coefficients of dissimilarity between the other specific ADE and the control group (Fig. 5B). The dominant bacteria at the genus level (top 35) for specific ADE treatments were *Lactobacillus*, *Subdoligranulum*, *Butyricicoccus*, *Lachnospiraceae*, *Bacteroides*, and *Candidatus Arthromitus*, while the EX and CTL groups trended higher in relative abundance for *Streptococcus* and *Enterococcus* (Fig. S3). The ileal microbial diversity index was remarkably (*P* < 0.05) positively correlated with soluble xylose, SAX, X2, X3, X5, and T-XOS contents by the clustering heatmaps (Fig. 5E). These soluble sugars and XOS components were analogously critical for ten families of bacteria (*P* < 0.05), including Ruminococcaceae, Lachnospiraceae, Butyricicoccaceae, Bacillacea, Christensenellacea, and Erysipelotrichaceae (Fig. 6A). Soluble sugars and oligosaccharides distinguished the communities along the first coordinate axis (34.48%), which was specifically embodied as higher contributions of SAX, X3, X5, and T-XOS to the microbial diversity (Fig. 6B). Moreover, glycan variables (SAX, SNSP, IAX, and INSP) explained 46.48% of the variance based on VPA analysis. However, the XOS produced by specific enzyme treatments explained 43.28% of the community changes, of which 35.39% could be explained by the induction of glycan dependence (Fig. 6C).

### Specific ADE triggered the production of major SCFAs in the cecal site of broiler chickens

**Fig. 7:**
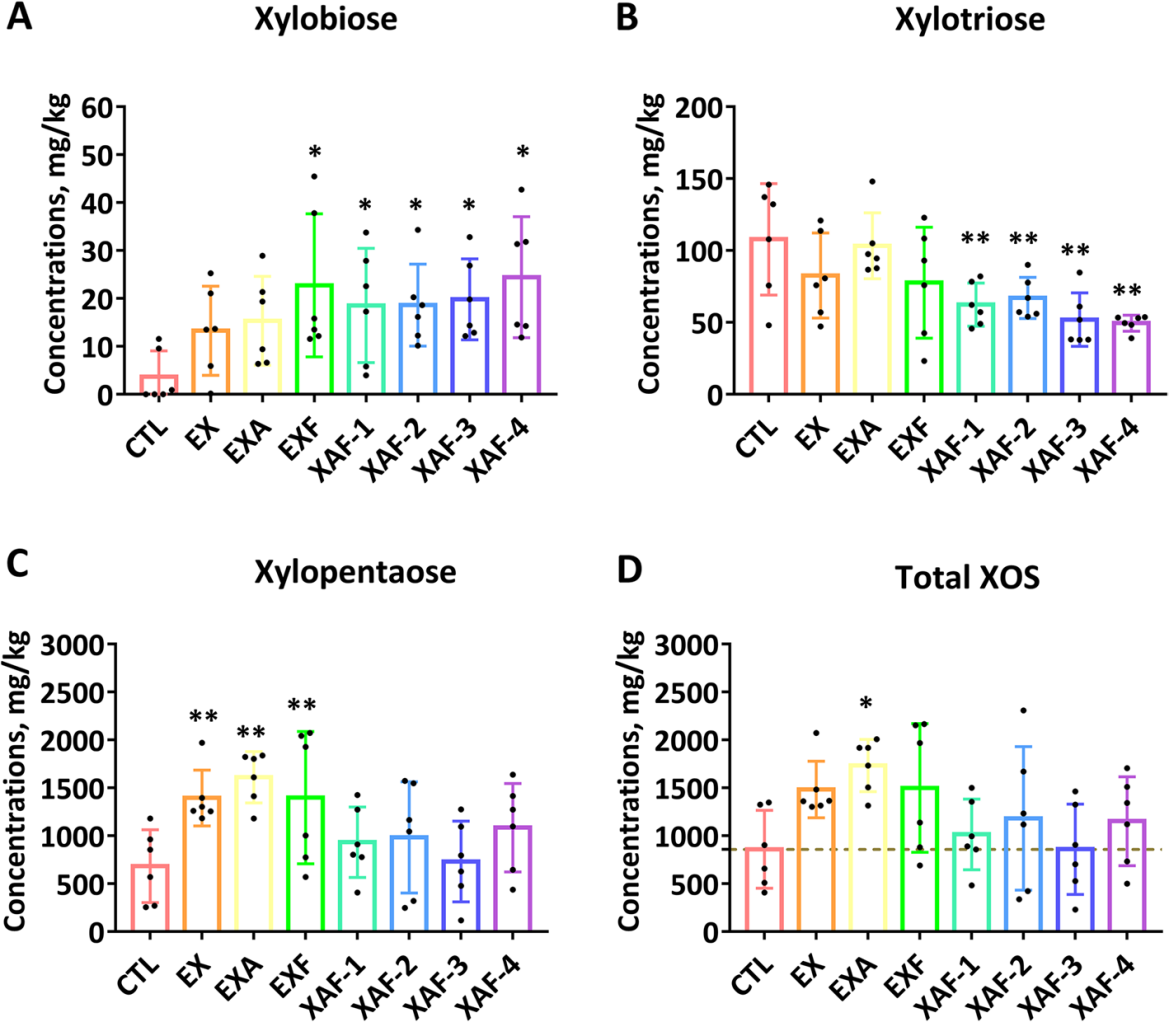
XOS yield in broiler chickens cecal chyme treated with specific arabinoxylan-degrading enzymes. Various XOS components, such as xylobiose (**A**), xylotriose (**B**), xylopentaose (**C**), and total XOS (**D**). Bars labeled with asterisks are significantly different (^*^P < 0.05, ^**^P < 0.01) compared with CTL. Data are expressed as the means ± SEM (*n* = 6)

**Fig. 8:**
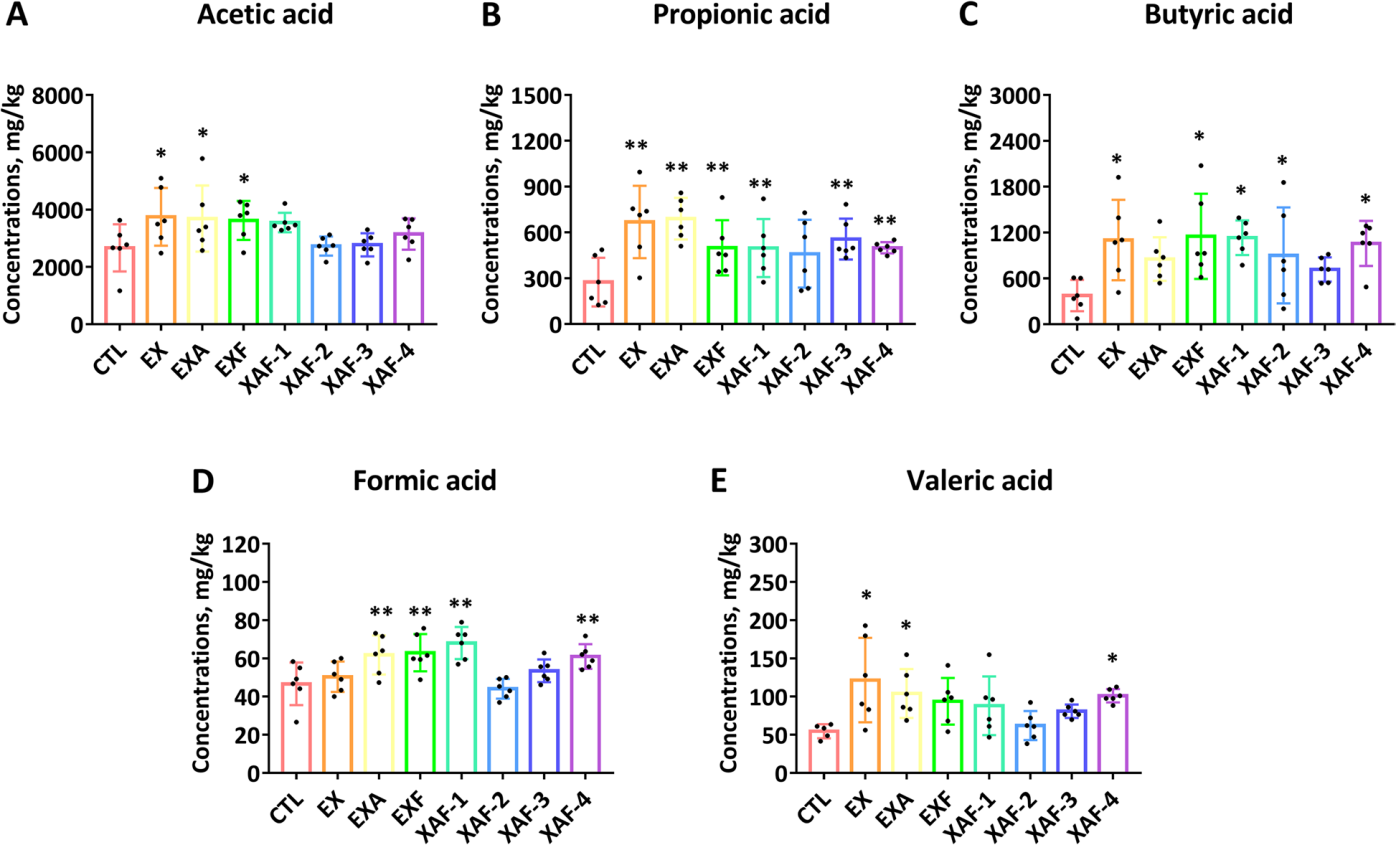
Specific arabinoxylan-degrading enzyme supplementation increased the major SCFA levels in the caecum of broiler chickens. **A** Acetic acid; **B** propionic acid; **C** butyric acid; **D** formic acid; **E** valeric acid. Bars labeled with asterisks are significantly different (^*^P < 0.05, ^**^P < 0.01) compared with CTL. Data are expressed as the means ± SEM (*n* = 6)

Unlike the specific dominant relationship of XOS in the posterior ileum, the contents of X3 in the ceca were decreased in all enzymatic treatments (Fig. 7B), and the corresponding changes in XAF were significant (*P* < 0.01). The contents of X2 in the EXF and XAF groups were increased significantly (*P* < 0.05) (Fig. 7A). Compared with the CTL group, the contents of X5 in the ceca of all ADE groups were increased (Fig. 7C), and the promotion effects of EX, EXA, and EXF were the most significant (*P* < 0.01). This was consistent with the changes in the total amount of XOS in the ceca (*P* < 0.05) (Fig. 7D), while X4 and X6 components were not detected in chyme samples. As shown in Fig. 8, the production of acetic acid, butyric acid, and propionic acid was dominant in the ceca of 21-day-old broiler chickens, while the contents of formic acid and valeric acid were extremely low. Specifically, the concentrations of acetic acid were significantly increased in the EX, EXA, and EXF groups (Fig. 8A), and the contents of butyric acid and propionic acid were significantly increased in most ADE groups (*P* < 0.05) (Fig. 8B, C). According to Spearman’s correlation test (Fig. S4), formic acid and butyric acid were significantly positively correlated with X5, X6, and TXOS in the ileum, and acetic acid was significantly positively correlated with BWG in 21-day-old broiler chickens (*P* < 0.05). However, isobutyric acid was significantly negatively correlated with X2 and goblet cell counts (*P* < 0.05). The branched clustering tree on the left side of Fig. S4 intuitively reflects the close relationships between sugar composition, gut development, and growth performance regulated by SCFAs.

### Beneficial microbes ameliorated ileal development and promoted the growth performance of broiler chickens

**Fig. 9:**
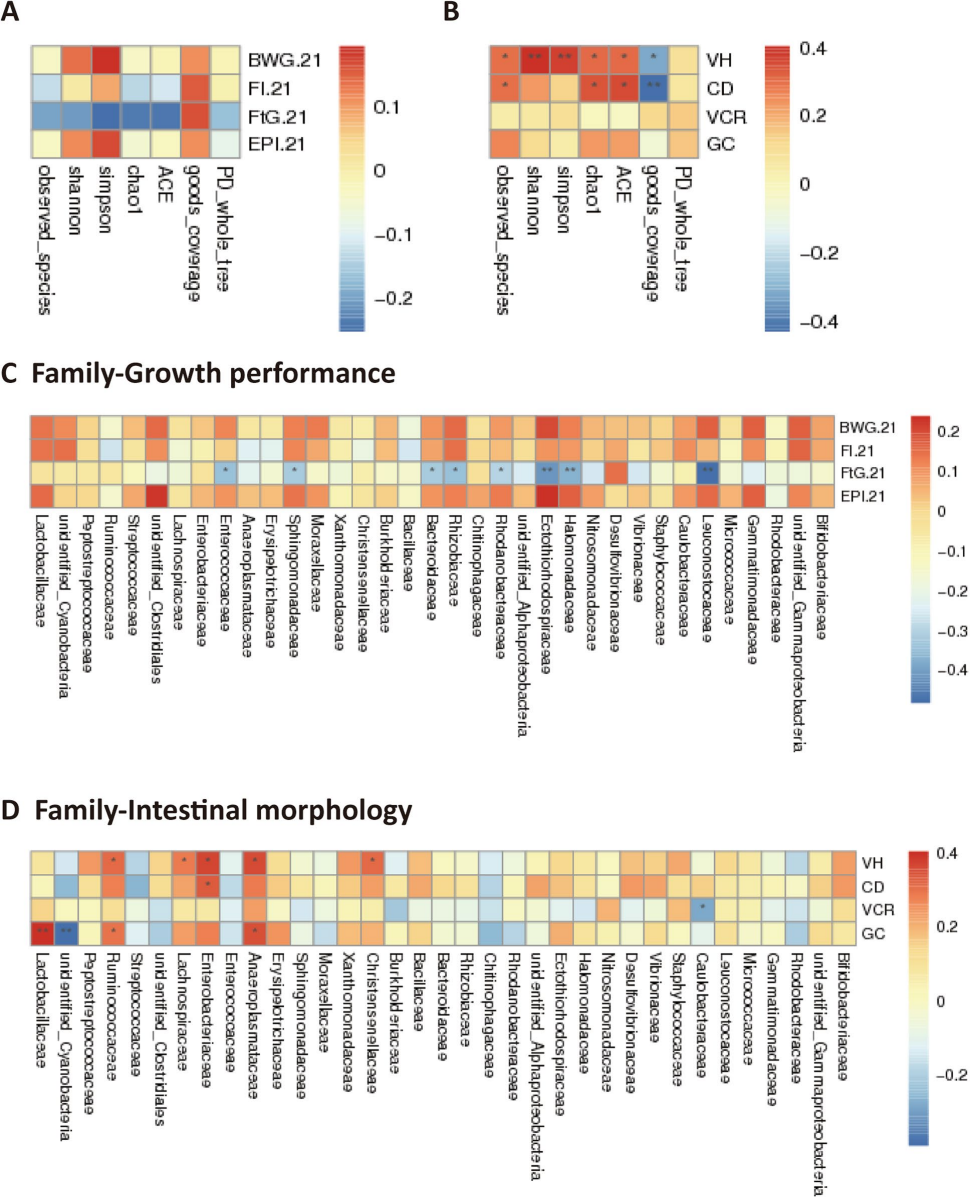
Correlation analysis integrating the ileal microbiome and intestinal morphology or growth performance. The intensity of the colors represents the degree of association based on Spearman’s correlation coefficients (red, positive correlation; blue, negative correlation; ^*^P < 0.05, ^**^P < 0.01)

Dietary EXF treatment had significant effects (*P* < 0.05) on the BWG of broiler chickens from d 14 to 21, and it tended to increase (*P* < 0.10) the feed conversion efficiency and EPI of broiler chickens at 5–21 d (Table 3). As shown in Fig. 9B, VH and CD were significantly positively correlated with the diversity and abundance of ileal microbes (*P* < 0.05). In addition, the longer intestinal villi were closely related to Lachnospiraceae, Ruminococcaceae, Anaeroplasmataceae, and Christensenellaceae (*P* < 0.05). Lactobacillaceae effectively (*P* < 0.01) stimulated the secretory functions of goblet cells (Fig. 9D). The F/G of broiler chickens had strikingly (*P* < 0.05) negative correlations with Enterococcaceae, Bacteroidaceae, and Sphingomonadaceae (Fig. 9C). Microbes with noteworthy differences (*P* < 0.05) were filtered to construct co-occurrence networks (Fig. S5). This demonstrated that community interactions were richer and more complex, and the multiplication of species was modified by *Lactobacillus* after EXF supplementation, which was critical to elevating the growth of broiler chickens (Table 3).

**Table 3:** Effects of specific arabinoxylan-degrading enzymes on growth performance of broiler chickens^1^

Items	BWG, g	FI, g	F/G, g/g	EPI	MRT, %
Phase 1 (d 14 to 21)					
CTL	466.53^b^	632.27	1.355	929.70	0.00
EX	469.45^ab^	633.11	1.371	932.16	1.39
EXA	456.82^b^	637.35	1.374	907.06	0.00
EXF	486.15^a^	644.08	1.327	988.55	0.00
XAF-1	462.57^b^	630.08	1.353	931.74	2.78
XAF-2	467.67^b^	631.73	1.326	940.83	1.39
XAF-3	465.08^b^	629.63	1.354	915.86	1.39
XAF-4	473.08^ab^	627.56	1.327	928.83	2.78
SEM	2.192	3.539	0.006	6.920	0.554
*P*-value	0.044	0.971	0.137	0.136	0.824
Phase 2 (d 5 to 21)					
CTL	789.08	1045.78	1.325	391.36	0.00
EX	799.65	1065.37	1.332	389.20	1.39
EXA	796.05	1057.27	1.328	387.52	1.39
EXF	831.80	1076.85	1.305	420.42	0.00
XAF-1	795.80	1058.14	1.339	387.21	4.17
XAF-2	795.35	1049.67	1.323	396.60	1.39
XAF-3	804.93	1063.23	1.323	400.86	1.39
XAF-4	796.18	1054.51	1.325	400.50	2.78
SEM	4.753	6.150	0.003	2.857	0.590
*P*-value	0.478	0.958	0.065	0.055	0.717

^1^Values are given as the means based on six birds for each treatment (*n* = 6). The abbreviations of the groups are consistent with those in Table 2

*BWG* Body weight gain, g; *FI* Feed intake, *g; F/G* Feed-to-gain ratio, g/g; *EPI* European comprehensive production index; *MRT* Mortality, %; *SEM* Standard error of the mean

^a,b^ Means in a column with superscripts without a common letter differ (*P* < 0.05)

## Discussion

Intestinal villi are the main sites of nutrient absorption, and their structural integrity and functional manifestations greatly affect the growth performance and overall health of animals. Long villi and shallow crypts are generally interrelated to support wider contact surface areas for higher absorption capacities and healthy development of the intestine [20, 21], but they also support greater tissue turnover, accounting for the optimal status of the gut. The current study showed that most specific ADE combinations significantly increased the jejunal VH and VCR and reduced the corresponding CD at 21 d, which was similar to a previous report by Wang et al. [22]. Moreover, microscopic analysis and statistical quantification of the villi also indicated that ADE supplements resulted in more goblet cells in both the jejunum and ileum of broiler chickens, thereby synthesizing and secreting lubricant mucus to protect the small intestine [23]. It has been demonstrated that the digestive and absorptive functions of the small intestine are closely related to morphological changes in VH and CD [21, 22]. In this study, as expected, dietary ADE treatments had significant effects on maltase activities in the ileal mucosa and upregulated Na^+^-K^+^ ATPase activities in the small intestine mucosa at 21 d in broiler chickens. Thus, the enhanced intestinal growth might improve absorption abilities or nutrient utilization and further benefit the subsequent growth performance.

AX in corn comprises the majority of the NSP in this grain tissue. It is frequently distinguished with reference to water solubility, an important benchmark for enzymatic hydrolysis, while corn contains less-soluble AX [24]. On the other hand, the abundance of arabinose residues, along with attachments decorated with various linkages, is key to its multifarious and complex character. Of the phenolic cross-links in corn AX, ferulic acid makes up 90% or more of the total and comprises 5% of the cell wall [25]. These covalent linkages offer strength and rigidity to structures with lower solubility, which may trigger another way to impede access to the primary xylan backbone. It is reasonable to think that debranching enzymes, such as EA and EF, could work in collaboration with main chain enzymes to increase enzymatic accessibility and to improve the efficiency of core EX. The positive impacts of ADE supplements could be explained by their ability to generate prebiotic XOS from dietary AX [26]. In this study, chromatographic technology was used to track the ileal chyme of broiler chickens fed corn-type diets containing specific ADE to highlight the conversion characteristics of corn AX and its product components. The data showed that specific ADEs were responsible for the increased solubilization of IAX or INSP in the ileum of enzyme-fed broiler chickens. Recently, the term “kick-starter” has been proposed to suggest that XOS could form a signaling agent for microbiota to boost the development of their fiber-degrading capacity in young broilers [27]. This provides evidence for our findings that higher levels of IAX solubilization in the ileum were also reported even though these oligosaccharides were generated in a small quantity. There were dissimilarities in the types of XOS products in the terminal ileum corresponding to the different specific ADE combinations. The results indicated that synergistic and sequential removal of substituents allowed for more rapid degradation of corn AX. Hence, it proves once more that the pattern of released XOS reaffirms a chronological process - the removal of branched components is followed by xylan backbone degradation [7, 28].

XOS, sugar oligomers produced by xylan enzymatic hydrolysis, are considered emerging or candidate prebiotics. The beneficial effects of XOS on performance characteristics and the overall host health status have been well documented in the last decade and are well known for their prebiotic potential to broilers [29, 30]. Among the explicit functions of XOS, the selective stimulation of beneficial bacterial populations is the most vital demonstration and manifestation. In the present study, it was demonstrated that the microbial richness and diversity produced by specific ADE groups (EXA, EXF, XAF-1, and XAF-2) significantly increased, suggesting that their effects on the composition of the ileal microbial community were more prominent. The results were consistent with the findings of Wang et al., who reported that a xylanase supplementation mixture modified the overall structure of the fecal bacterial community in terms of β-diversity, with observably distinctive OTUs appearing [21]. Evidently, positive correlations between ileal microbial diversity indexes and XOS also indicated that the oligosaccharide products were essential in early nutrition to stimulate and establish gut microbiota in the broiler chickens intestine [31, 32]. The dominant bacteria at the genus level for specific ADE treatments were *Lactobacillus*, *Butyricicoccus*, *Lachnospiraceae*, and *Bacteroides*, and this observation was in line with many recent reports [23, 33, 34]. These probiotics could lead to the competitive exclusion of pathogenic bacteria and benefit the gut health of broilers [35]. However, the differential flora enriched in the ileum was dissimilar for each specific ADE treatment. The variable results may be attributed to the fact that the effects of multienzyme complexes containing debranching enzymes on XOS production are inseparable from their associated cleavage properties and the order of action when bound to EX [28]. In brief, different ADE combination types yielded specific XOS components in the ileum of broiler chickens, thereby promoting the selective proliferation of beneficial bacteria. SAX, X2, X3, X5, and T-XOS, for instance, were critical for ten families of bacteria, such as Ruminococcaceae, Lachnospiraceae, Christensenellaceae, and Butyricicoccaceae. Collectively, the conversion of corn AX to assorted XOS under the ADE treatments benefited microbial homeostasis.

The formation of XOS in the lower intestinal tract is considered a benefit of AX degradation, acting as prebiotics for anaerobic bacteria in the ceca for energy and other purposes [14]. The common microbes in the hindgut, including *Bifidobacterium* and *Lactobacillus*, are conducive to ferment AX hydrolysis products to SCFAs (mainly acetate, propionate and butyrate) [14]. A wealth of data indicate that SCFAs are superior energy sources for intestinal epithelial cells and for their role as signaling molecules to modulate gut integrity and immune response [36]. In addition, these microbial metabolites fermented from complex carbohydrates play a major role in microbiota-host interactions. Thus, the composition of XOS and the contents of SCFA in the ceca were also monitored to clearly assess the dynamic changes in the posterior intestinal segment of broiler chickens. Herein, the markedly increased concentrations of X2, X5, and T-XOS in the cecal chyme after most specific ADE treatments were associated with subsequent SCFAs production. It was also demonstrated that the concentrations of acetic acid, butyric acid, and propionic acid were significantly advanced in most ADE groups, suggesting that supplementation-specific ADE combinations effectively promoted the production of XOS in the hindgut segment of broiler chickens and fermentation to output SCFAs.

Although there is no shortage of studies indicating that exogenous xylanase escalated the nutrient digestibility and growth performance of broilers fed corn basal diets, few specific enzyme mixtures, including debranching enzymes or their transformative effects in vivo, were not clear [23, 34, 37, 38]. In the current study, improvements in growth performance with supplementation with EXF might be ascribed to the beneficial bioactivities of feruloylated XOS via prebiotic, immunomodulatory, and/or antioxidant effects [39]. This discrepancy could be explained by the complex community interactions, with the multiplication of species modified by *Lactobacillus* after EXF supplementation. Additionally, to further understand how specific ADE-induced changes in the gut microbiota modulate host health, Spearman association analysis was used to expound potential impacts on the growth and development of broiler chickens. The higher VH was significantly positively correlated with the diversity and abundance of ileal microbes (Lachnospiraceae, Ruminococcaceae, and Lactobacillaceae), revealing the internal factors of intestinal morphology and microflora. Similarly, a previous study concluded that *Lactobacillus* enrichment was positively correlated with ileal VH and lactate concentration, and *Enterobacter*, *Staphylococcus*, and *Pseudomonas* were negatively associated with intestinal development [23]. Accordingly, it was speculated that the specific EXF might effectively connect corn AX, product XOS, microbial community, and physical health to playing prebiotic roles.

## Conclusions

In summary, specific xylanase supplementation of corn basal diets was capable of improving the broiler chickens growth performance. Xylanase mixtures potentially advanced the conversion output of corn AX to XOS. Such improvements were necessarily associated with positive shifts in the microbial community, intestinal morphology, ileal absorptive capacity, and intracecal fermentation. Therefore, it is recommended that debranching enzymes be used in future studies of fabricating prebiotic XOS in broiler basal diets.

## Supplementary Information

**Additional file 1: Fig. S1.** Diagram of the proportion of specific ADE treatments in the respective XOS components.

**Additional file 2: Fig. S2.** Specific arabinoxylan-degrading enzyme supplementation shifted the ileal microbiota community of broiler chickens.

**Additional file 3: Fig. S3.** Ileal microbial community distribution heatmap at the genus level (top 35).

**Additional file 4: Fig. S4.** Correlation of SCFA components in the hindgut of broiler chickens with multiple indicators.

**Additional file 5: Fig. S5.** Co-occurrence network analysis of ileal microbiota at the genus level for EXF supplementation.

## Data Availability

The datasets used and/or analysed during the current study are available from the corresponding author on reasonable request.

## Abbreviations

ADEs: Arabinoxylan-degrading enzymes
AX: Arabinoxylan
CTL: Control treatment group
DDGS: Corn distillers dried grains with solubles
EA: Arabinofuranosidase
EF: Ferulic acid esterase
EX: Endo-xylanases
NSPs: Nonstarch polysaccharides
OTU: Operational taxonomic units
PCoA: Principal coordinates analysis
RDA: Redundancy analysis
SCFA: Short-chain fatty acids
SEM: Standard error of the mean
XOS: Xylooligosaccharides
