# Supplementary material for: Debranching enzymes decomposed corn arabinoxylan into xylooligosaccharides and achieved prebiotic regulation of gut microbiota in broiler chickens
Source: J Anim Sci Biotechnol. 2023 Mar 9;14:34. doi: 10.1186/s40104-023-00834-3 (PMC9996988; doi:10.1186/s40104-023-00834-3)
Supplement: Supplementary file 1 — Additional file 1: Fig. S1. Diagram of the proportion of specific ADE treatments in the respective XOS components. [file 40104_2023_834_MOESM1_ESM.docx]

**
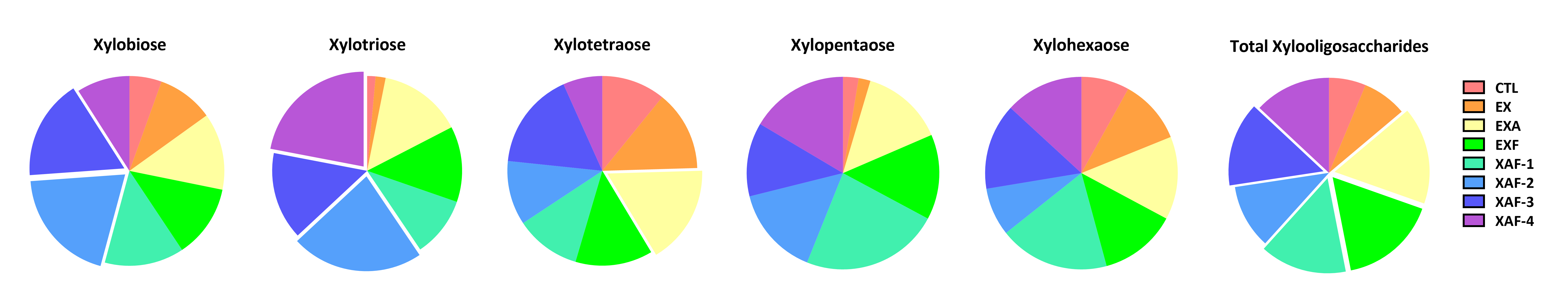
**

**Fig. S1** Diagram of the proportion of specific arabinoxylan-degrading enzymes treatments in the respective XOS components**.** The proportion of color block in the pie chart was determined by the ratio of each enzyme treatment content to the total content of corresponding oligosaccharides
