# Supplementary material for: Debranching enzymes decomposed corn arabinoxylan into xylooligosaccharides and achieved prebiotic regulation of gut microbiota in broiler chickens
Source: J Anim Sci Biotechnol. 2023 Mar 9;14:34. doi: 10.1186/s40104-023-00834-3 (PMC9996988; doi:10.1186/s40104-023-00834-3)
Supplement: Supplementary file 2 — Additional file 2: Fig. S2. Specific arabinoxylan-degrading enzyme supplementation shifted the ileal microbiota community of broiler chickens. [file 40104_2023_834_MOESM2_ESM.docx]

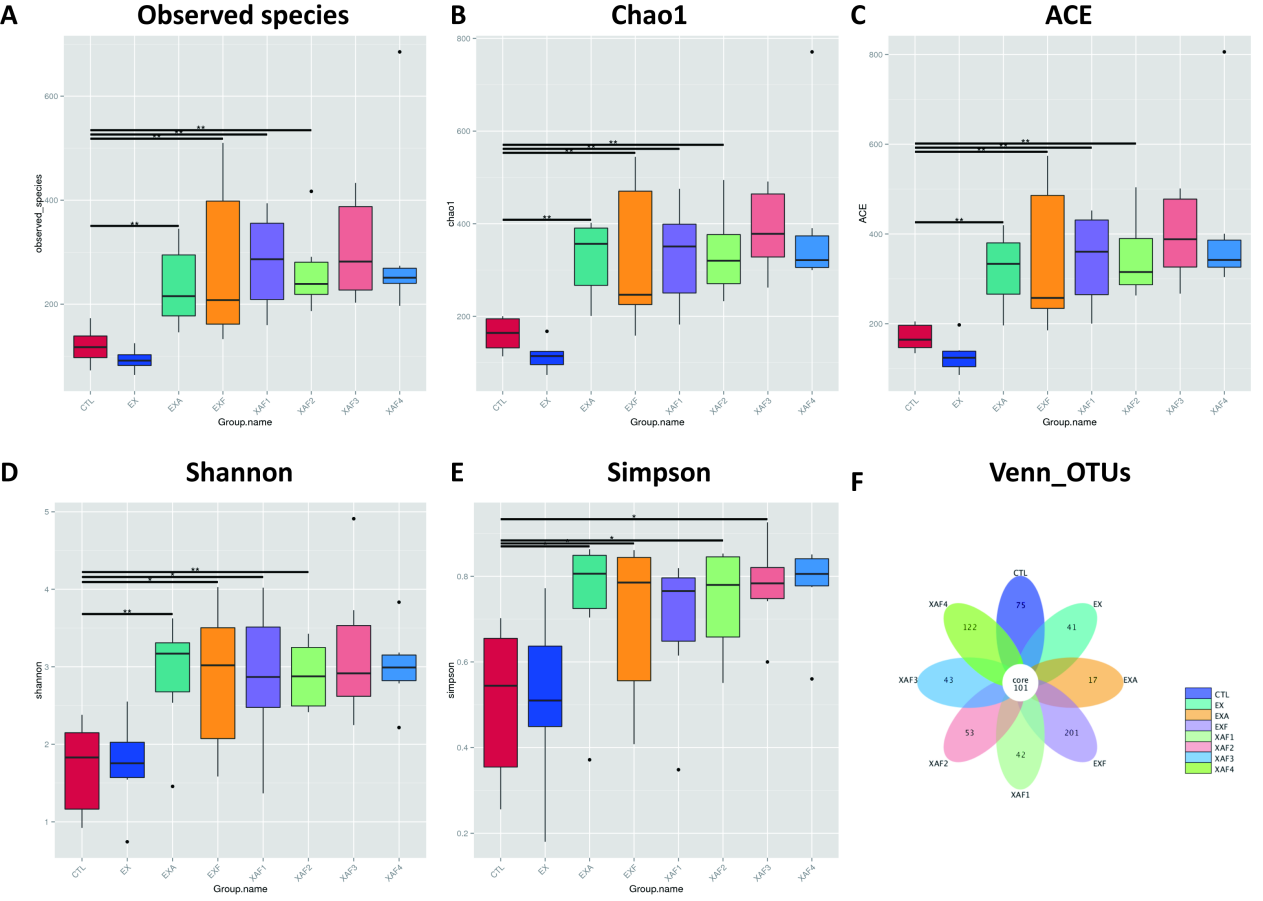


**Fig. S2** Specific arabinoxylan-degrading enzyme supplementation shifted the ileal microbiota community of broiler chickens. The bacterial richness estimated by observed species (**A**), Chao1 (**B**), and ACE (**C**), as well as its evenness estimated by Shannon and Simpson index (**D**, **E**). The box plots intuitively reflected the species diversity and whether the differences were significant through the Wilcoxon rank-sum test (^*^ *P* < 0.05, ^**^ *P* < 0.01)
