# Supplementary material for: Debranching enzymes decomposed corn arabinoxylan into xylooligosaccharides and achieved prebiotic regulation of gut microbiota in broiler chickens
Source: J Anim Sci Biotechnol. 2023 Mar 9;14:34. doi: 10.1186/s40104-023-00834-3 (PMC9996988; doi:10.1186/s40104-023-00834-3)
Supplement: Supplementary file 3 — Additional file 3: Fig. S3. Ileal microbial community distribution heatmap at the genus level (top 35). [file 40104_2023_834_MOESM3_ESM.docx]

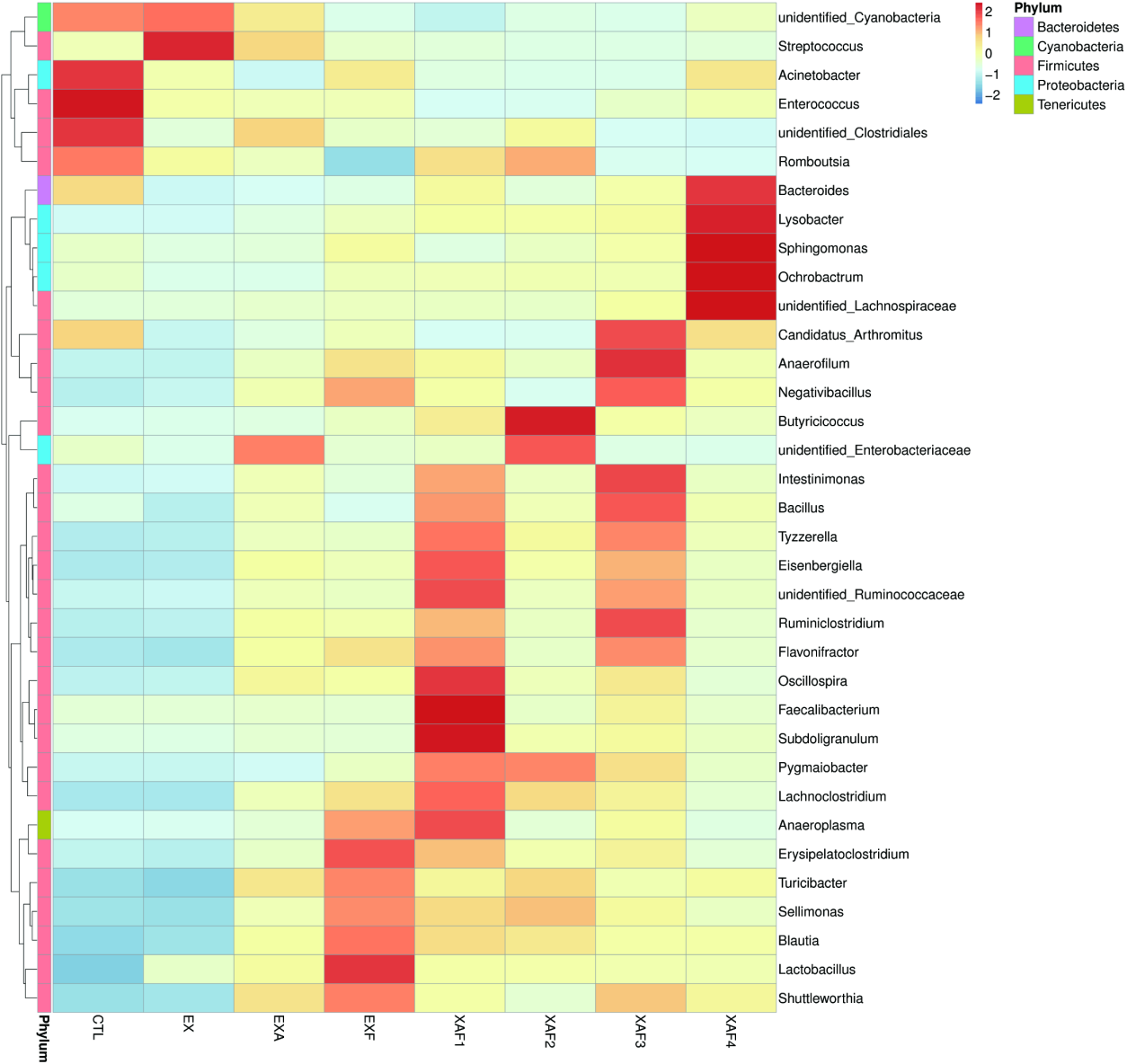


**Fig. S3** Ileal microbial community distribution heatmap at the genus level. Based on the species annotations and abundance information of all samples at the genus level, we selected the top 35 genera in abundance and clustered them both at the species and sample levels. The vertical direction in the figure revealed the sample information, the horizontal direction showed the species annotation, and the cluster tree on the left of the figure denoted the species cluster tree
