# Supplementary material for: Debranching enzymes decomposed corn arabinoxylan into xylooligosaccharides and achieved prebiotic regulation of gut microbiota in broiler chickens
Source: J Anim Sci Biotechnol. 2023 Mar 9;14:34. doi: 10.1186/s40104-023-00834-3 (PMC9996988; doi:10.1186/s40104-023-00834-3)
Supplement: Supplementary file 4 — Additional file 4: Fig. S4. Correlation of SCFA components in the hindgut of broiler chickens with multiple indicators. [file 40104_2023_834_MOESM4_ESM.docx]

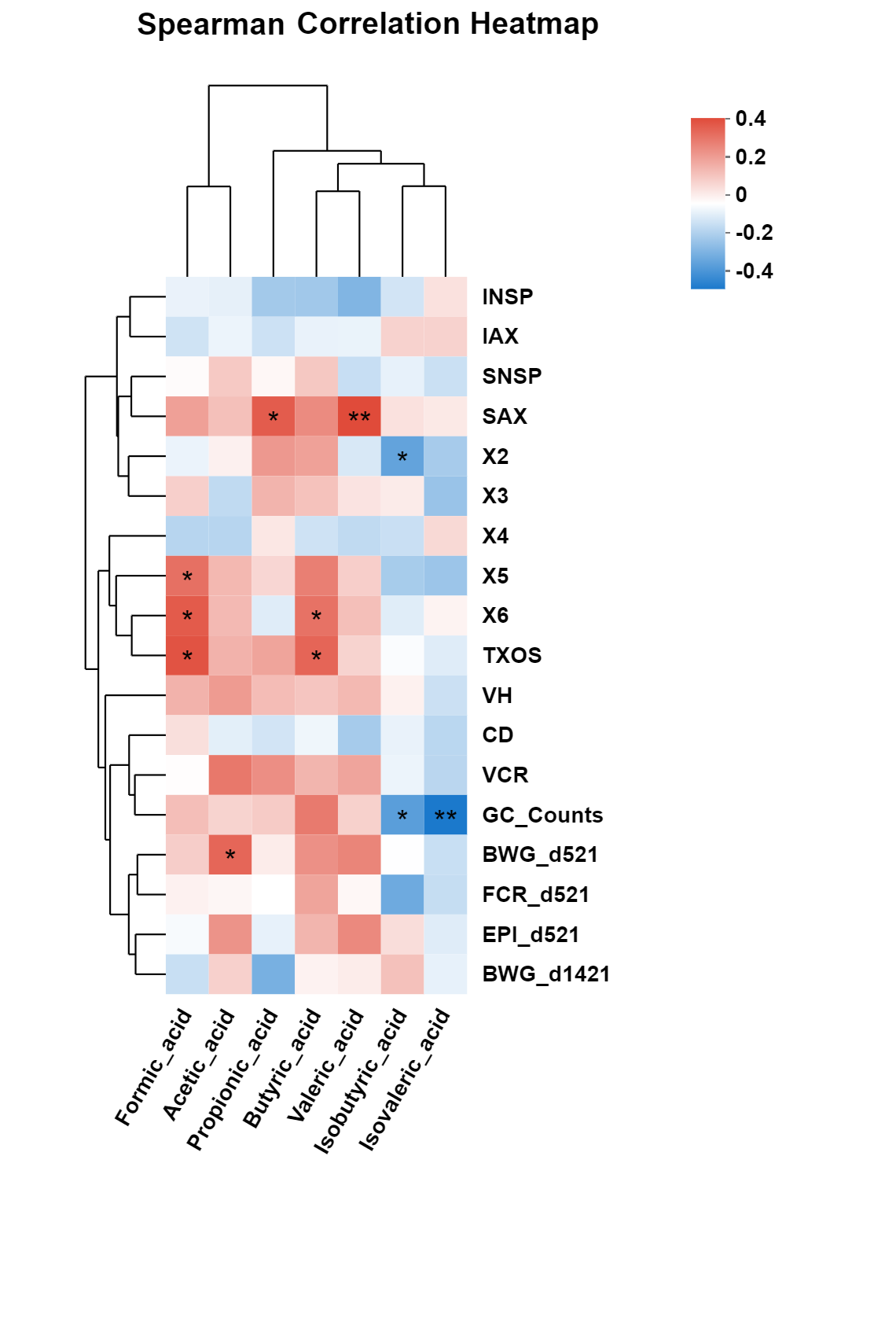


**Fig. S4** Correlation of SCFA components in the hindgut of broiler chickens with multiple indicators. The intensity of the colors represents the degree of association based on spearman’s correlation coefficients (red, positive correlation; blue, negative correlation; ^*^*P* < 0.05, ^**^*P* < 0.01)
