# Supplementary material for: Debranching enzymes decomposed corn arabinoxylan into xylooligosaccharides and achieved prebiotic regulation of gut microbiota in broiler chickens
Source: J Anim Sci Biotechnol. 2023 Mar 9;14:34. doi: 10.1186/s40104-023-00834-3 (PMC9996988; doi:10.1186/s40104-023-00834-3)
Supplement: Supplementary file 5 — Additional file 5: Fig. S5. Co-occurrence network analysis of ileal microbiota at the genus level for EXF supplementation. [file 40104_2023_834_MOESM5_ESM.docx]

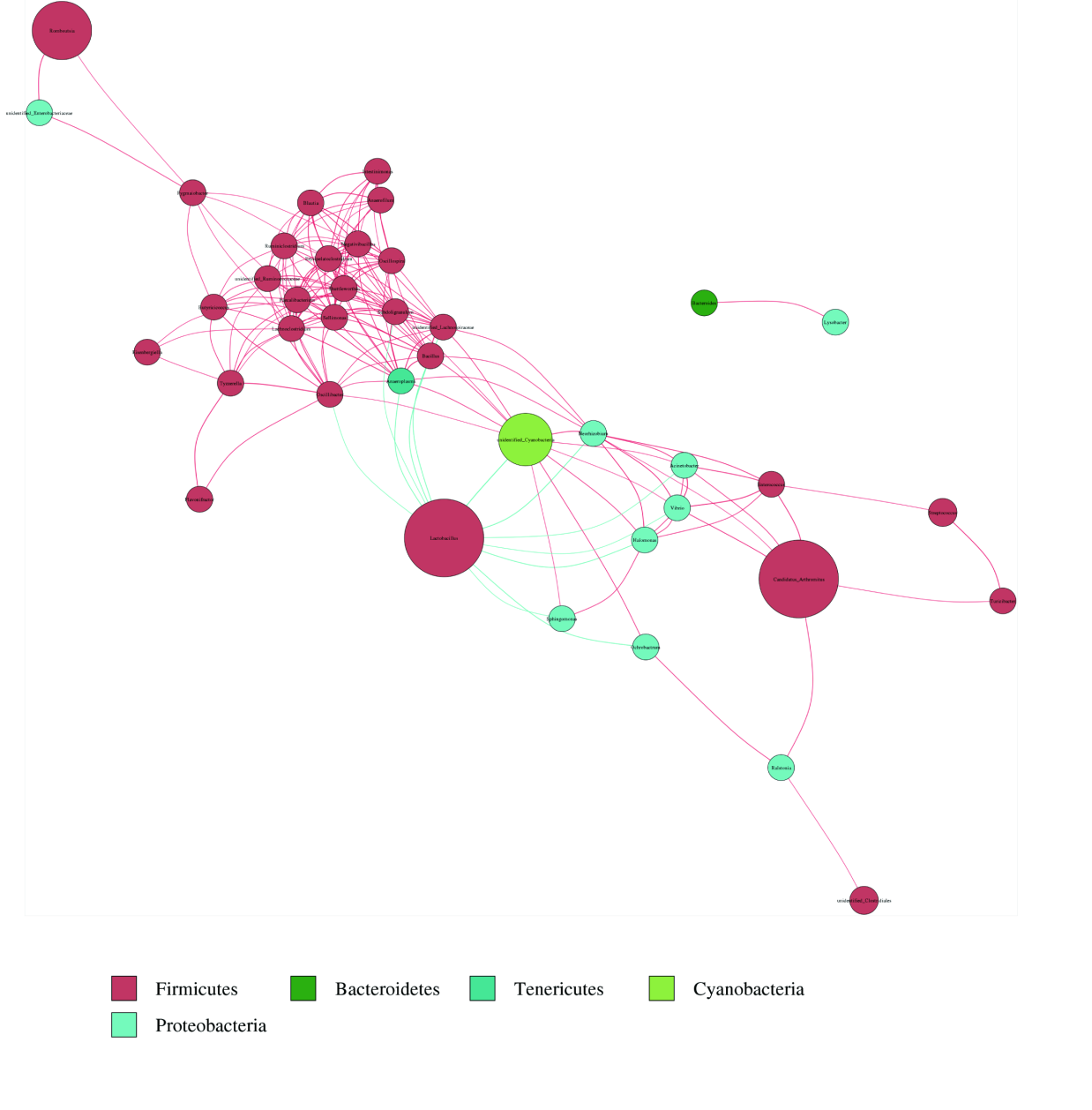


**Fig. S5** Co-occurrence network analysis of ileal microbiota at the genus level for EXF supplementation. Nodes represent different genera, and their sizes show the average relative abundance of the genus. The thickness of the line between nodes is positively relational with the absolute values of the correlation coefficients (red, positive correlation; blue, negative correlation)
